# 467. Antifungal Therapy in *Candida* Intra-abdominal Infections Following Source Control

**DOI:** 10.1093/ofid/ofac492.525

**Published:** 2022-12-15

**Authors:** Margaret Buck, Dustin R Carr, Derek N Bremmer, Daniel Jenniches, James Babowice, Eunice L Chung

**Affiliations:** Allegheny General Hospital, Pittsburgh, Pennsylvania; Allegheny General Hospital, Pittsburgh, Pennsylvania; Allegheny General Hospital, Pittsburgh, Pennsylvania; Allegheny General Hospital, Pittsburgh, Pennsylvania; Allegheny General Hospital, Pittsburgh, Pennsylvania; Allegheny General Hospital, Pittsburgh, Pennsylvania

## Abstract

**Background:**

Current literature suggests that many surgical patients with *Candida* isolated from an intra-abdominal source do not develop symptomatic infections, however, Infectious Disease Society of America (IDSA) Guidelines recommend antifungal treatment in *Candida* intra-abdominal infections. An article published by Fabre et al found similar incidence of treatment failure between patients appropriately treated for *Enterococcus* intra-abdominal infections compared to patients without *Enterococcus* coverage following source control. *Enterococcus* and *Candida* are both low-virulent microorganisms, questioning the necessity of antifungal therapy following source control in *Candida* intra-abdominal infections.

**Methods:**

This was a retrospective, cohort study of adult patients with intra-abdominal infections who underwent source control and isolation of *Candida* in intra-abdominal cultures. Eligible patients were separated based on receipt of antifungals. Patients were excluded if they received between 24 hours and 3 days of antifungal therapy, had candidemia prior to intervention, or received prophylactic or therapeutic antifungals within 7 days of intervention. The antifungal group and non-antifungal group were assessed for incidence of treatment failure, defined as death or additional unplanned surgical or antimicrobial interventions for intra-abdominal infections.

**Results:**

A total of 125 patients were included with 77 patients (61.6%) receiving antifungal therapy and 48 patients (38.4%) not receiving antifungal therapy. Patients who received antifungal therapy had a higher median SAPS II score (29 vs 22.5; p = 0.003). There was no difference in the incidence of treatment failure at 30 days between the antifungal and non-antifungal groups (41.6% and 35.2%, respectively; p = 0.62). Median hospital length of stay (14 vs 8.5 days; p < 0.001) and median total duration of antimicrobial therapy (10 vs 7 days; p = 0.667) was longer in the antifungal group compared to the non-antifungal group, respectively.

**Conclusion:**

Patients with *Candida* intra-abdominal infections that underwent source control had a similar rate of treatment failure at 30 days regardless of receipt of antifungal therapy. However, those who received antifungal therapy had a higher severity of illness.

**Disclosures:**

**Dustin R. Carr, PharmD, BCPS, BCIDP**, Merck: Honoraria **Derek N. Bremmer, PharmD, BCPS-AQ ID**, Thermo Fisher Scientific: Honoraria.

